# *ZmNAC17* Integrates Transcriptional and Protein Interaction Networks to Regulate Maize Stalk Architecture

**DOI:** 10.3390/plants15121814

**Published:** 2026-06-12

**Authors:** Tianyu Yang, Ming Wang, Haiyan Zhang, Qiuhua Li, De Xue, Jinjie Guo, Fuchao Jiao, Jingtang Chen

**Affiliations:** 1 College of Agronomy, Qingdao Agricultural University, Qingdao 266109, China; 2 Shandong Key Laboratory of Maize Biological Breeding, Qingdao 266109, China; 3 The Characteristic Laboratory of Crop Germplasm Innovation and Application, Qingdao 266109, China; 4 Zibo Boxin Agricultural Technology Co., Ltd., Zibo 256408, China; 5 College of Agronomy, Hebei Agricultural University, Baoding 071001, China

**Keywords:** maize, plant height, *ZmNAC17*, transcriptome, phytohormones

## Abstract

Maize plant height and stalk mechanical strength are critical traits that influence planting density, yield, and lodging resistance. Although numerous dwarf mutants have been characterized in maize, most cannot be directly utilized in breeding programs due to associated developmental and reproductive deficiencies. In a previous study, we demonstrated that *ZmNAC17* regulates mesocotyl elongation by mediating auxin and reactive oxygen species (ROS) biosynthetic pathways. Here, we characterize the role of *ZmNAC17* in maize stalk development using both *zmnac17* mutants and *ZmNAC17*-overexpressing (OE) lines. Plant height, stalk diameter, and internode length were reduced in both the *zmnac17-1* EMS mutant and the *zmnac17-3* CRISPR mutant. Internode cell length and cell area were decreased, whereas cell number was increased in *zmnac17-1*. Cellulose and lignin contents were elevated in *zmnac17-1*. Stalk bending force was diminished in *zmnac17-3* but enhanced in the OE lines. The ratio of syringyl to guaiacyl (S/G), a key lignin monomer composition, was increased in *zmnac17-3* while reduced in the OE lines. ZmNAC17 functions as a transcription factor, with its downstream targets implicated in phytohormone biosynthesis, phytohormone signaling, and lignin biosynthesis. CUT&Tag binding profile, EMSA, and dual-luciferase reporter assay demonstrate that ZmNAC17 promotes the expression of *caffeoyl-CoA O-methyltransferase* (*CCoAOMT*). IP-MS, Co-IP, and GST pull-down assays reveal that ZmNAC17 interacts with Beta glucosidase aggregating factor1 (BGAF1). Collectively, our findings indicate that *ZmNAC17* regulates maize stalk development through transcriptional activation and protein–protein interactions, thereby providing new genetic resources for modifying plant architecture and mechanical strength in maize.

## 1. Introduction

Maize (*Zea mays* L.) is one of the most widely grown crops throughout the world. Increasing yield has always been a primary goal in maize breeding. Over the last few decades, the improvement of maize yield was largely dependent on the rise in planting density [1]. However, high planting density increases the risk of lodging. Reducing plant height is important in both improving resistance to lodging and reducing the cost of field management [2]. Although a large number of maize dwarf mutants have been characterized, few of them can be applied straightforwardly in breeding, because of developmental and reproductive defects [3]. The regulation mechanism of plant height development is complex and far from elucidated [4]. Therefore, identifying novel dwarf mutants is important in improving maize plant stature.

Plant height is controlled by the biosynthesis and signaling of phytohormones, including gibberellic acid (GAs), auxin (IAAs), and brassinosteroid (BRs) [5,6,7]. The famous “Green Revolution” genes *Semidwarfing1* (*SD1*) and *Reduced height-1* (*Rht-1*) are both related to the GA pathway. Wheat *Rht-1* encodes DELLA, a key negative regulator of GA signal transduction [8]. Rice *SD1* encodes GA20ox, which is a critical enzyme in GA biosynthesis [9,10,11]. Maize mutants affecting GA biosynthesis and signaling have also been reported. *D1* encodes a GA 3-oxidase catalyzing the final step of bioactive GA synthesis [3]. *D3* encodes a cytochrome P450 enzyme involved in the early stages of GAs biosynthesis [12]. *D8* and *D9* encode DELLA protein; *d8* and *d9* mutants all show a dwarf phenotype [13]. Early-stage GA biosynthesis and GA signaling caused an extensive maize dwarf phenotype and developmental deficiency. Currently, the most frequently used dwarf mutant in maize is *brachytic2* (*br2*). *BR2* is mainly expressed in the vascular bundles of the node and internode. *br2* suppresses the elongation of lower internodes for excessive auxin accumulation in the intercalary meristem region [6,14].

The regulation of plant phytohormone homeostasis is complex and involves transcription factors. Transcription factors have been experimentally demonstrated to negatively regulate plant height. The maize NAC transcription factor family comprises over 100 members with documented roles in growth regulation and reproductive development. For example, overexpression of OsNAC2 reduces rice plant height by inhibiting the expression of ent-kaurene oxidase 2 (KO2), which is an early step enzyme in GA biosynthesis [15]. High expression of OsNAC129 decreases rice plant height through the BR pathway [16]. A transcriptional hierarchy governs secondary cell wall (SCW) deposition. Tissue-specific NAC domain proteins, including SND1, NST1/2, and VND6/7, activate MYB46 and MYB83 transcription factors. MYB46 and MYB83 bind to the SMRE cis-element and turn on the biosynthetic genes for cellulose, xylan, and lignin, thereby orchestrating the entire SCW program [17]. Overexpressing OsEATB, a member of AP2/ERF transcription factor, reduces rice plant height by downregulating OsCPS2, which encodes the first step of the GA biosynthetic enzyme [18]. OsbZIP49 negatively regulates rice plant height by affecting the content of IAA by regulating OsGH3-2 and OsGH3-13 [19]. High expression of ZmMADS3 decreases the number of maize nodes and affects spikelet development with unclear mechanisms [20]. Some transcription factors are found to have a role in regulating plant architecture. For instance, ZmIBH1-1, encoding a basic helix-loop-helix (bHLH) transcription factor, functions as a negative regulator of maize leaf angle [21].

Because few maize dwarf mutants have been applied successfully in breeding, it is of importance to identify novel genes regulating stalk development. Previously, we found that *ZmNAC17* (Zm00001eb185110) regulates maize mesocotyl elongation by mediating auxin and ROS biosynthetic pathways [22]. The mesocotyl length in *zmnac17-1* was lower than that in B73. The identified DEGs between zmnac17-1 and B73 were mainly enriched in the “tryptophan metabolism” and “antioxidant activity” pathways. *zmnac17-1* exhibited a decrease in the content of indole acetic acid (IAA) and an increase in the content of reactive oxygen species (ROS). In this study, we comprehensively characterize the function of *ZmNAC17* in maize stalk development, encompassing plant height, ear development, grain quality, and stalk mechanical strength. We demonstrate that *ZmNAC17* is a nuclear-localized transcriptional activator that directly regulates target genes involved in cell wall biosynthesis and forms protein complexes with cell wall-associated regulators. Furthermore, we integrate phytohormone profiling, transcriptome analysis, and molecular validation to elucidate a multi-layered regulatory network. Our results provide new insights into the molecular mechanisms of *ZmNAC17* in maize stalk development and offer promising targets for improving lodging resistance.

## 2. Results

### 2.1. Phenotypic Characterization of the Maize zmnac17-1 and zmnac17-3 Mutants

**Figure 1:**
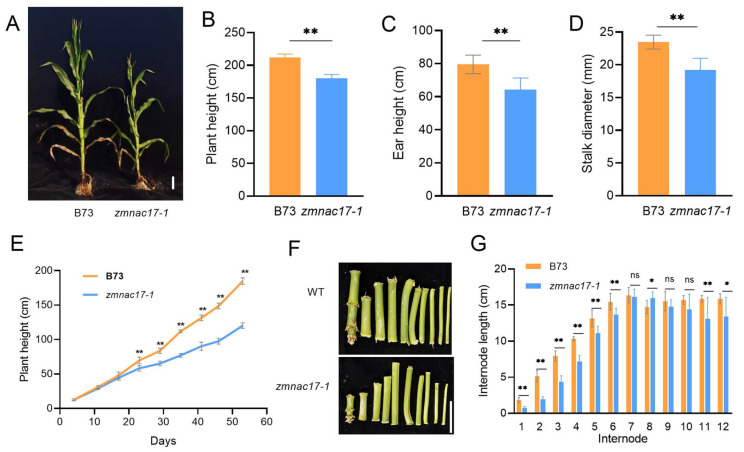
Plant height of WT (B73) and *zmnac17-1* (EMS mutant). (**A**) Photos of plant height of B73 and *zmnac17-1*. Bar = 20 cm. (**B**–**D**) Plant height, ear height, and stalk diameter of B73 and *zmnac17-1*, respectively. (**E**) Growth curves of B73 and *zmnac17-1*. (**F**) Photos of internodes of B73 and *zmnac17-1*. Bar = 10 cm. (**G**) Internode length of B73 and *zmnac17-1*. (* *p* < 0.05, ** *p* < 0.01, ns, not significant).

To investigate the function of *ZmNAC17* in maize plant height development, we grew the *zmnac17-1* mutant in the field. The plant height in the wild-type (WT) B73 was about 212 cm, while in *zmnac17-1* it was about 180 cm. The ear height in WT was about 80 cm, but in *zmnac17-1* was about 64 cm. The third stalk internode diameter in WT was about 23 mm; however, in *zmnac17-1*, it was about 19 mm. Compared with WT, plant height, ear height, and the third internode diameter in *zmnac17-1* were reduced by about 15%, 19%, and 18%, respectively (Figure 1A–D). The plant height difference between WT and *zmnac17-1* can be observed from 16 days after seedling emergence, at which stage the plant height in WT was 47 cm, while in *zmnac17-1* it was about 44 cm. The difference in plant height reached a maximum at 40 days after seedling emergence, at which stage the plant height in WT reached 131 cm, while in *zmnac17-1* it was only about 90 cm (Figure 1E). There was no difference in node number between WT and *zmnac17-1*. The length of most internodes was longer in WT than in *zmnac17-1*, especially in the first to the sixth internodes. There was no difference in the length of the seventh, the 10th, and the 11th internode (Figure 1F,G).

**Figure 2:**
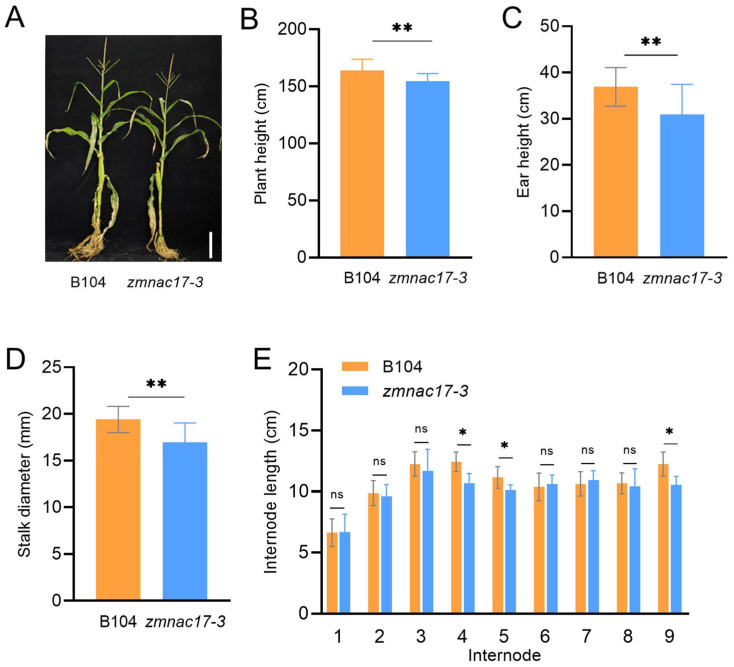
Plant height of WT (B104) and *zmnac17-3* (CRISPR mutant). (**A**) Photos of plant height of WT (B104) and *zmnac17-3*. Bar = 20 cm. (**B**–**D**) Plant height, ear height, and stalk diameter of WT (B104) and *zmnac17-3*, respectively. (**E**) Internode length of WT (B104) and *zmnac17-3*. The data presented are means ± SD and statistically calculated by Student’s unpaired *t*-test (* *p* < 0.05, ** *p* < 0.01, ns, not significant).

To further confirm the function of *ZmNAC17* in plant height regulation, we generated *zmnac17-3* using CRISPR/Cas9 gene editing in the B104 background. Consistent with *zmnac17-1*, *zmnac17-3* also showed significantly reduced plant height, ear height, and stalk diameter compared to the wild-type B104. The internode elongation pattern of *zmnac17-3* was similar to that of *zmnac17-1* (Figure 2). Despite the different genetic backgrounds and mutagenesis methods, both mutants exhibited consistent phenotypes, including reduced plant height, ear height, and internode length, confirming that the observed effects are specifically attributable to *ZmNAC17* deficiency. These results demonstrate that *ZmNAC17* plays a role in regulating maize plant height.

**Figure 3:**
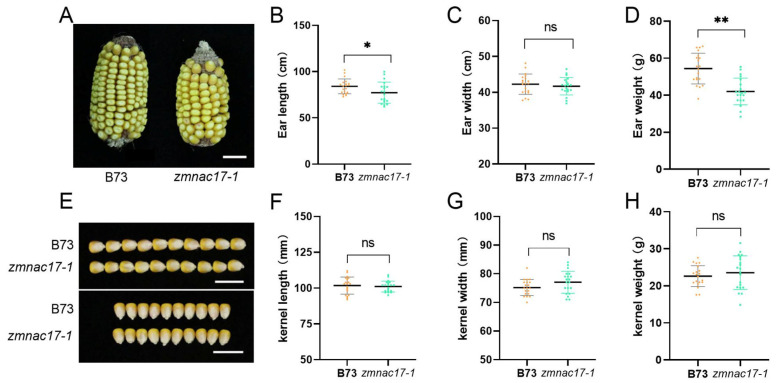
Ear and kernel development in WT (B73) and *zmnac17-1*. (**A**) Photos of ears of WT and *zmnac17-1*. Bar = 2 cm. (**B**–**D**) Ear length, ear width, and ear weight of WT and *zmnac17-1*, respectively. 20 biological replicates were used. The data presented are means ± SD and statistically calculated by Student’s unpaired *t*-test (* *p* < 0.05, ** *p* < 0.01, ns, not significant). (**E**) Photos of kernels of WT and *zmnac17-1*. Bars = 2 cm. (**F**–**H**) 10-kernel length, 10-kernel width, and 100-kernel weight of WT and *zmnac17-1*, respectively. 20 biological replicates were used. The data presented are means ± SD and statistically calculated by Student’s unpaired *t*-test (* *p* < 0.05, ** *p* < 0.01, ns, not significant).

It has been reported that most maize dwarf mutants have developmental and reproductive deficiency [2,3,6]. In order to know how reproductive ability was affected in *zmnac17-1*, we measured kernel yield-related traits. The ear length was about 84 cm and 77 cm in WT and *zmnac17-1*, respectively (Figure 3A). The ear weight was about 54 g and 42 g in WT and *zmnac17-1*, respectively. The ear length and ear weight were reduced by 8% and 22% in *zmnac17-1* than those in WT, respectively (Figure 3B,D). There was no difference for ear width (Figure 3C), kernel length (Figure 3E,F), kernel width (Figure 3G), and 100-kernel weight between *zmnac17-1* and WT (Figure 3H).

**Figure 4:**
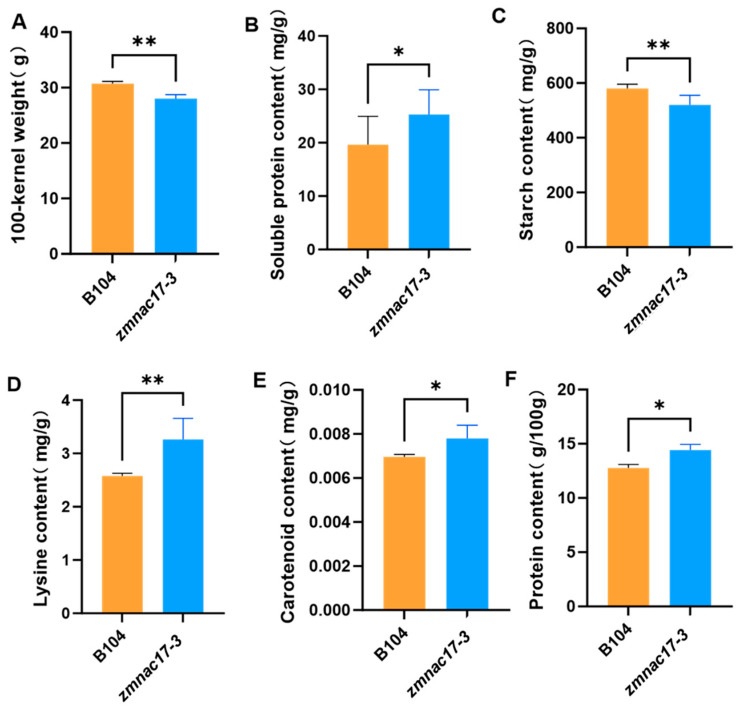
Kernel weight and nutritional quality in WT (B104) and *zmnac17-3* (CRISPR mutant). (**A**) 100-kernel weight. (**B**) Soluble protein content. (**C**) Starch content. (**D**) Lysine content. (**E**) Carotenoid content. (**F**) Total protein content determined by the Kjeldahl method. 20 biological replicates were used for 100-kernel weight (**A**), and 3 biological replicates for kernel quality analysis (**B**–**F**). The data presented are means ± SD and statistically calculated by Student’s *t*-test (* *p* < 0.05, ** *p* < 0.01).

In contrast to *zmnac17-1*, where 100-kernel weight was unaffected (Figure 3H), *zmnac17-3* showed significantly reduced 100-kernel weight (Figure 4A). Furthermore, kernel nutritional quality was markedly altered in *zmnac17-3*, with increased soluble protein, lysine, and carotenoid contents but decreased starch content (Figure 4B–E). Total protein content was significantly increased in *zmnac17-3* compared to WT (Figure 4F), consistent with the elevated soluble protein content (Figure 4B). These results indicate that *ZmNAC17* deficiency affects grain nutrient metabolism in a genetic background-dependent manner.

### 2.2. Cellular and Chemical Characterization of zmnac17-1

**Figure 5:**
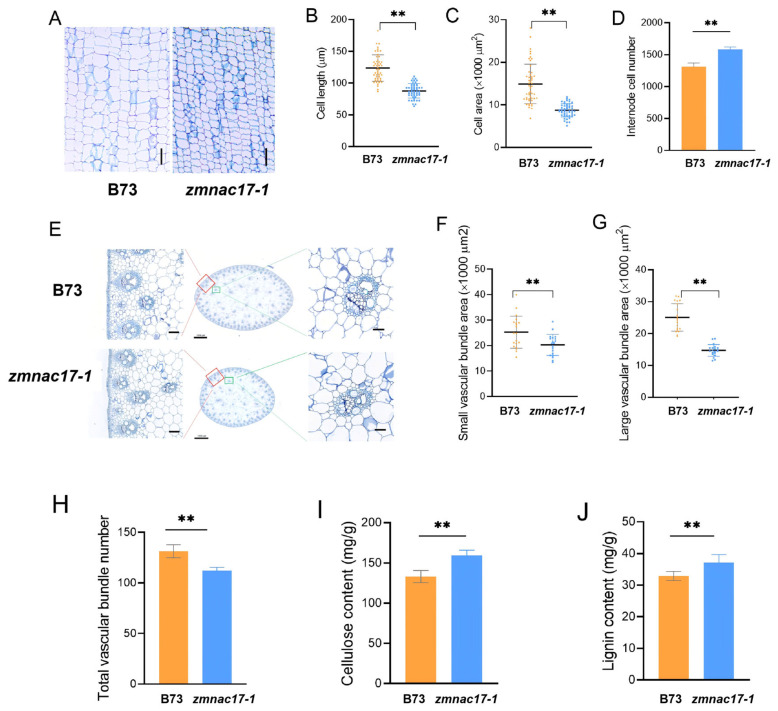
Cellular characterization of WT (B73) and *zmnac17-1*. (**A**) Photos of longitudinal sections of the sixth internode stems. Bars = 200 μm. (**B**,**C**) Cell length, cell area, and internode cell number of WT and *zmnac17-1*, respectively. In total, 50 biological replicates were used. (**D**) Internode cell number of WT and *zmnac17-1*. Internode cell number was calculated based on the internode length and cell length. Three biological replicates were used. (**E**) Photos of cross sections of tassel stems. Bar = 100 μm, 1000 μm, 50 μm, from left to right, respectively. (**F**,**G**) Small vascular bundle area and large vascular bundle area of WT and *zmnac17-1*, respectively. In total, 20 biological replicates were used. (**H**) Total vascular bundle number of WT and *zmnac17-1*. Three biological replicates were used. (**I**,**J**) Cellulose and lignin content of WT and *zmnac17-1*, respectively. Three biological replicates were used. The data presented are means ± SD and statistically calculated by Student’s unpaired *t*-test (** *p* < 0.01 ).

Maize at the tasseling stage is sensitive to lodging; the strength of the third to the sixth internode has been used to indicate lodging resistance [23]. We have known that the internode is shorter in *zmnac17-1* than that in WT. To test how the *zmnac17-1* plant internode is influenced at the cellular level, we performed microscopic observation using the longitudinal sections of the sixth internode (Figure 5A). We found that the cell length of WT and *zmnac17-1* was about 123 µm and 87 µm, respectively (Figure 5B). Cell area of WT and *zmnac17-1* was 0.015 µm^2^ and 0.009 µm^2^, respectively (Figure 5C). The cell length and cell area of the sixth internode decreased by 28.97% and 41.36% in *zmnac17-1* than those in WT, respectively. Contrarily, the internode cell number increased 18% in *zmnac17-1* than that in WT (Figure 5D).

Vascular bundle size and number are closely related to maize stalk strength [24]. To know whether the vascular bundles differ between WT and *zmnac17-1*, we initially examined the transverse section using the sixth internode. However, we could not make a high-quality transverse section due to technical problems, so we used the tassel stem instead (Figure 5E). Compared with WT, the average area of small and large vascular bundles decreased by 19.83% and 41.31% in *zmnac17-1*, respectively (Figure 5F and Figure 5G). The total number of vascular bundles decreased by 14.50% in *zmnac17-1* (Figure 5H). The reduced number of vascular bundles and area in *zmnac17-1* was consistent with its decreased stem diameter.

Cellulose and lignin are the main components of the secondary cell wall [25]. To know whether these components differ between *zmnac17-1* and WT, we measured their contents using the sixth internode seven days after pollination, which is often used in indicating maize logging resistance ability [24]. The cellulose and lignin contents in WT were about 133 mg/g and 33 mg/g, respectively (Figure 5I), while in *zmnac17-1* were about 160 mg/g and 37 mg/g, respectively (Figure 5J). Compared with WT, the contents of cellulose and lignin in the third internode of *zmnac17-1* increased by 19.89% and 12.66%, respectively. The increase in cellulose and lignin contents in *zmnac17-1* was consistent with the rise in cell number.

**Figure 6:**
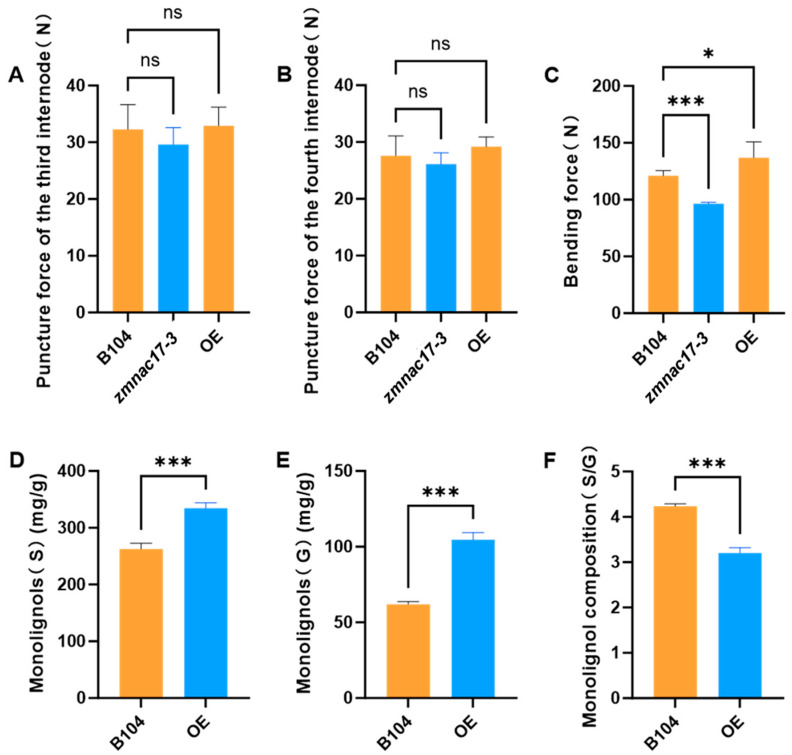
Stalk mechanical strength and lignin monomer composition in WT (B104) and *ZmNAC17* transgenic lines. (**A**) Rind penetration resistance of the third internode. (**B**) Rind penetration resistance of the fourth internode. (**C**) Bending force of the third internode. In total, 15 biological replicates were used for (**A**–**C**). (**D**) S-type lignin monomer content. (**E**) G-type lignin monomer content. (**F**) S/G ratio. OE1 and OE2 represent two independent overexpression lines; KO1 and KO2 represent two independent knockout lines. Three biological replicates were used for (**D**–**F**). The data presented are means ± SD and statistically calculated by Student’s *t*-test for (**A**–**C**) ( * *p* < 0.05, *** *p* < 0.001, ns, not significant), and by one-way ANOVA with Dunnett’s multiple comparison test for (**D**–**F**) (*** *p* < 0.001).

To assess stalk mechanical properties, we measured rind penetration resistance and bending force in the B104 background at the R3 stage (18–22 days after silking). The bending force of the *zmnac17-3* mutant was significantly reduced by approximately 21% compared to WT, whereas rind penetration resistance showed no significant difference (Figure 6A–C). Notably, the magnitude of the bending force reduction exceeded that of the stem diameter reduction, implying that *ZmNAC17* influences intrinsic cell wall mechanical properties beyond morphological changes.

Since lignin content and monomer composition are key determinants of stalk mechanical strength, we further analyzed lignin monomers in both knockout and overexpression (OE) lines. Lignin monomer composition analysis revealed that in the OE lines, both S-type and G-type lignin monomer contents were significantly increased, while the syringyl/guaiacyl (S/G) ratio was significantly decreased from 4.24 to approximately 3.14 (Figure 6A–C). Conversely, *zmnac17-3* showed a slightly increased S/G ratio. Given that G-type monomers form more C–C bonds (e.g., β-5 and 5-5 linkages) and contribute to a more compact lignin polymer network, the reduced S/G ratio in OE lines is consistent with their enhanced bending resistance. These results demonstrate that *ZmNAC17* positively regulates stalk mechanical strength by optimizing lignin monomer composition.

### 2.3. Subcellular Localization and Transcriptional Activity of ZmNAC17

**Figure 7:**
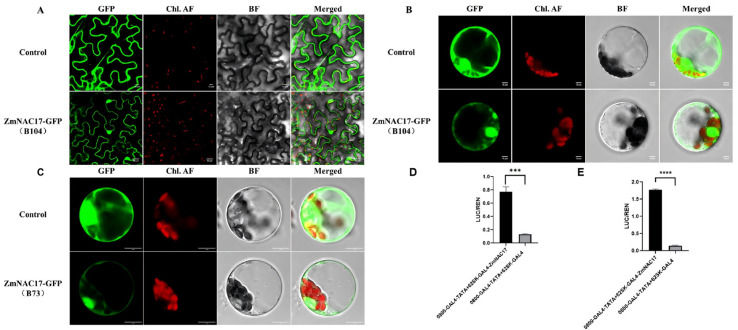
Subcellular localization and transcriptional activation activity of ZmNAC17. (**A**) ZmNAC17-GFP fusion protein in tobacco leaf epidermal cells. Scale bars = 10 μm. (**B**) ZmNAC17-GFP in B104 maize protoplasts. Scale bar = 10 μm. (**C**) ZmNAC17-GFP in B73 maize protoplasts. Scale bar = 10 μm. GFP, green fluorescent protein; Chl. AF, chlorophyll autofluorescence; BF, bright field; Merge, merged image. (**D**) Transcriptional activation activity at effector:reporter = 1:1. (**E**) Transcriptional activation activity at effector:reporter = 9:1. VP16 was used as a positive control. The data presented are means ± SD (*n* = 3 biological replicates) and statistically calculated by Student’s *t*-test (*** *p* < 0.001, **** *p* < 0.0001).

To investigate the molecular function of ZmNAC17, we examined its subcellular localization and transcriptional activity. The ZmNAC17-GFP fusion protein was predominantly localized to the nucleus in both tobacco leaf epidermal cells and maize protoplasts (B104 and B73 backgrounds), with only weak cytoplasmic signals observed (Figure 7A–C). No nuclear enrichment was detected in the GFP empty vector control. Furthermore, a dual-luciferase reporter assay in maize protoplasts confirmed that *ZmNAC17* possesses strong transcriptional activation activity. When the effector and reporter vectors were used at a 1:1 ratio, the LUC/REN ratio of the ZmNAC17 group was approximately 5.8-fold higher than that of the empty vector control (Figure 7D). When the ratio was optimized to 9:1, the activation fold increased to 12.7 (Figure 7E). These results establish ZmNAC17 as a nuclear-localized transcriptional activator, consistent with its function as an NAC transcription factor.

**Figure 8:**
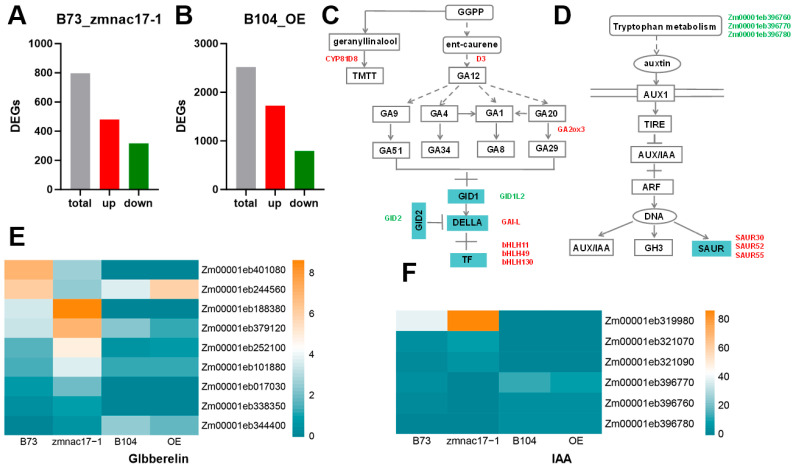
RNA-seq data for quantitative verification. (**A**,**B**) The number of DEGs. (**C**–**F**) DEGs involved in auxin and gibberellin synthesis and signal transduction, respectively.

To know the transcriptomic regulatory function of ZmNAC17, we performed RNA-seq using *zmnac17-1,* B73, ZmNAC17-HA overexpression (OE) lines, and B104. A total of 797 differentially expressed genes (DEGs) were detected between *zmnac17-1* and B73, with 480 genes up-regulated and 317 genes down-regulated in *zmnac17-1* (Figure 8 and Figure S1). A total of 2477 DEGs were identified between ZmNAC17-HA OE lines and B104. For the DEGs between *zmnac17-1* and B73, gene ontology (GO) enrichment analysis showed that the DEGs were significantly enriched in “photosynthesis”, “photosynthesis, light reaction”, and “regulation of hormone levels” GO terms (Figure S2). KEGG enrichment analysis showed that the DEGs were enriched in “Metabolic pathways”, “Biosynthesis of secondary metabolites”, “Plant-pathogen interaction”, “Plant hormone signal transduction”, “Photosynthesis”, and “Photosynthesis-antenna proteins” pathways (Figure S3). These results indicated that DEGs are related to various pathways. Some genes were further validated by qRT-PCR (Figure S4).

We found some DEGs involved in the biosynthesis and signal transduction of endogenous phytohormones (Figure 8, Table S1). For biosynthesis and signal transduction of Gibberellin, D3 (Zm00001eb379120), GA2ox3 (Zm00001eb338350), GAI-L (Zm00001eb344400), and three bHLH family genes were significantly up-regulated in *zmnac17-1*. GID1L2 (Zm00001eb244560) and GID2 (Zm00001eb401080) were down-regulated in *zmnac17-1*. In contrast, D3 (Zm00001eb379120) and GAI-L (Zm00001eb344400) were down-regulated in ZmNAC17-HA OE lines, and GID1L2 was up-regulated in ZmNAC17-HA OE lines. For biosynthesis and signal transduction of IAAs, three SAUR family genes (Zm00001eb319980, Zm00001eb321090, Zm00001eb321070) were up-regulated, three genes (Zm00001eb433600, Zm00001eb396770, Zm00001eb396780) participating in tryptophan metabolism were down-regulated in *zmnac17-1*. However, these 6 genes were not significantly changed in ZmNAC17-HA OE lines. In addition, some genes involved in cytokinin, brassinolide, jasmonic acid, abscisic acid, ethylene, and salicylic acid biosynthesis and signal transduction processes were also altered (Table S1). Some transcription factors (TF) play important roles in cell wall metabolism and stalk development, such as NAC, Golden2-like (G2-like), bZIP, myeloblastosis (MYB), and BHLH [23,24,25,26,27]. Therefore, we sorted out TF genes from the DEGs and found 49 TF DEGs (Table S2). Among them, eight NAC family genes, six G2-like family genes, and four bZIP family genes were up-regulated in *zmnac17-1*.

**Figure 9:**
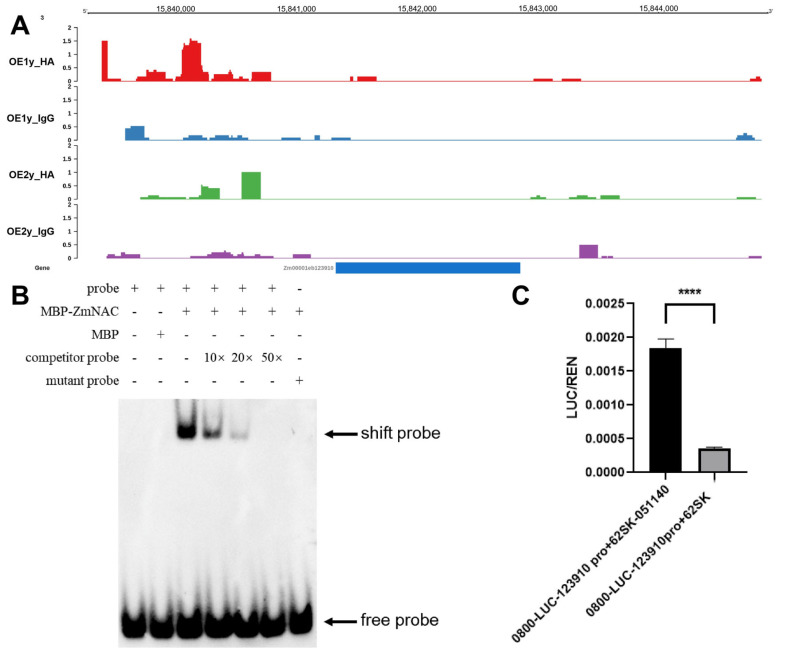
Validation of *ZmNAC17* binding to and activation of *CCoAOMT*. (**A**) CUT&Tag binding profile at *CCoAOMT*. The red box indicates the significant enrichment peak (fold enrichment = 4.14). (**B**) EMSA shows specific binding of MBP-*ZmNAC17* to the wild-type probe (WT). (**C**) Dual-luciferase reporter assay. The data presented are means ± SD (*n* = 3 biological replicates) and statistically calculated by Student’s *t*-test (**** *p* < 0.0001).

To identify downstream targets of ZmNAC17, we performed CUT&Tag using the same OE lines and wild-type B104. Integration of CUT&Tag binding data with RNA-seq data revealed candidate target genes that were both bound by ZmNAC17 and transcriptionally regulated. After applying stringent filtering criteria (peak located within the 2 kb promoter region and fold enrichment ≥ 4), a lignin biosynthetic gene encoding caffeoyl-CoA O-methyltransferase (CCoAOMT) was identified as a high-confidence direct target. CUT&Tag data showed a significant enrichment peak in the promoter region of *CCoAOMT* (fold enrichment = 4.14) (Figure 9A). Electrophoretic mobility shift assay (EMSA) confirmed that the purified MBP-*ZmNAC17* protein specifically bound to a biotin-labeled cis-element probe in the promoter of *CCoAOMT*; this binding was effectively competed by an excess of unlabeled wild-type probe but not by a mutant probe (Figure 9B). Moreover, a dual-luciferase reporter assay demonstrated that *ZmNAC17* significantly activated the promoter of *CCoAOMT*, with an activation fold of 5.1 (Figure 9C). Collectively, these results establish that *ZmNAC17* functions as a direct transcriptional activator of lignin biosynthetic genes.

### 2.4. Proteins Interacted with ZmNAC17

**Figure 10:**
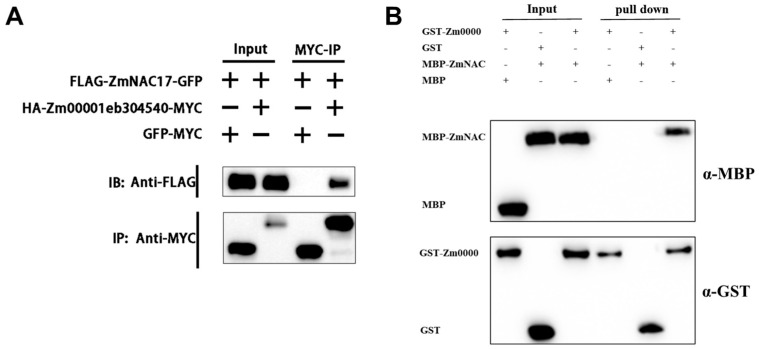
Validation of the interaction between ZmNAC17 and BGAF1. (**A**) Co-IP assay in maize protoplasts showing co-immunoprecipitation of BGAF1 with *ZmNAC17*. (**B**) GST pull-down assay showing direct interaction between MBP-*ZmNAC17* and GST-tagged BGAF1. Input, total protein extract; IP, immunoprecipitation. ‘+’ indicates presence, ‘−’ indicates absence.

Transcription factors often function through protein–protein interactions. To investigate whether ZmNAC17 forms complexes with other proteins to regulate cell wall metabolism, we performed immunoprecipitation coupled with mass spectrometry (IP-MS) using ZmNAC17-HA overexpression plants in the B104 background. Beta glucosidase aggregating factor1 (BGAF1, Zm00001eb304540) was identified as a high-confidence interacting protein (log_2_ fold enrichment = 6.56). BGAF1 contains a cell wall-associated protein containing a dirigent domain and a jacalin-related lectin domain. The interaction between ZmNAC17 and BGAF1 was further validated by co-immunoprecipitation (Co-IP) assays in maize protoplasts: BGAF1 was specifically co-immunoprecipitated with ZmNAC17 (Figure 10A). GST pull-down assays further demonstrated direct physical interaction between ZmNAC17 and BGAF1 in vitro (Figure 10B). These findings suggest that *ZmNAC17* may regulate cell wall biosynthesis through both transcriptional activation and protein complex formation.

### 2.5. The Plant Height of zmnac17-1 Can Be Regulated by GAs

**Figure 11:**
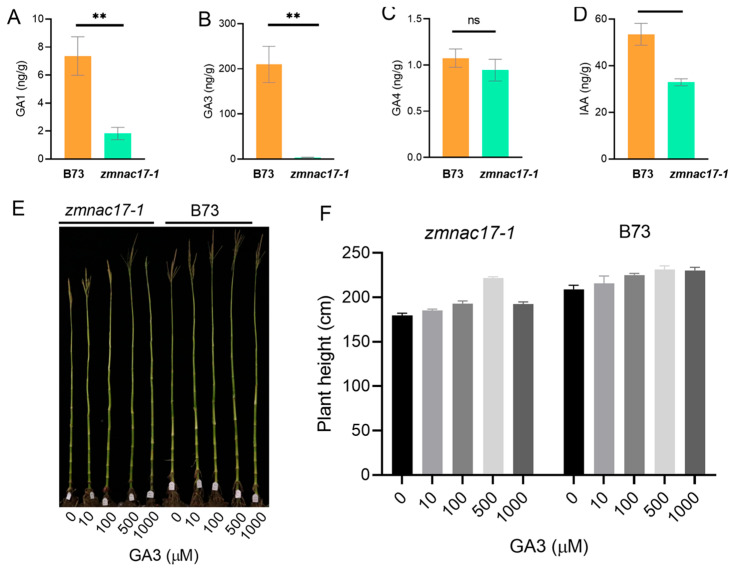
Plant endogenous phytohormone content and GA3 treatment in WT (B73) and *ZmNAC17*. (**A**–**D**) GA1, GA3, GA4, and IAA content in WT and *ZmNAC17*. (**E**) Photos of maize plants treated with different GA3 solutions. (**F**) Statistical analysis of maize plant height treated with different GA3 solutions. 3 biological replicates were used. The data presented are means ± SD and statistically calculated by Student’s unpaired *t*-test (** *p* < 0.01, ns, not significant).

Previous studies have shown that plant height is related to endogenous phytohormone content [7]. Our RNA-seq data have shown that some DEGs are involved in gibberellin and auxin biosynthesis and signaling pathways. To know whether the gibberellin and auxin contents in *zmnac17-1* have been decreased, we measured the contents of bioactive GA1, GA3, GA4, and IAA in the sixth internode of 40-day-old plants, at the same time as RNA-seq analysis. Interestingly, we found that GA3 content is 209.80 ng/g in WT, while it is only about 3.28 ng/g in *zmnac17-1* (Figure 11A). GA3 content was reduced 98% in *zmnac17-1*. GA1 content was 7.37 ng/g in WT, while it was 1.82 ng/g in *zmnac17-1* (Figure 11B). GA1 content decreased 75.30% in *zmnac17-1* compared with WT. GA4 content was 1.08 ng/g in WT and 0.95 ng/g in *zmnac17-1* (Figure 11C). There was no significant difference between WT and *zmnac17-1* for GA4 content. IAA content was 53.50 ng/g in WT but 32.92 ng/g in *zmnac17-1* (Figure 11D). IAA content was decreased by 38.47% in *zmnac17-1* than that of WT.

GA1 and GA3 are important in plant stem elongation. Since GA3 decreased 98% in *zmnac17-1*, to know whether GA3 can rescue the semi-dwarf phenotype of *zmnac17-1*, we sprayed WT and *zmnac17-1* plants with a series of GA3 solutions (0 µM, 10 µM, 100 µM, 500 µM, and 1000 µM, respectively). We found that spraying 10 µM GA3 slightly increased the plant height of *zmnac17-1* and WT by 2.91% and 6.22%, respectively (Figure 11E). When sprayed with 100 µM GA3, the plant height of *zmnac17-1* and WT increased by 7.46% and 7.65%, respectively (Figure 11E). When treated with 500 µM GA3, the plant height of *zmnac17-1* and WT increased by 23.05% and 10.53%, respectively. When treated with 1000 µM GA3, the plant height of *zmnac17-1* and WT increased by 6.90% and 10.05%, respectively (Figure 11F). The plant height of *zmnac17-1* reached a maximum when using 500 µM GA3. When treated with 500 µM GA3, the plant height of *zmnac17-1* was about 225 cm, while B73 was about 230 cm, and 500 µM GA3 partially alleviated the dwarf phenotype of *zmnac17-1*.

## 3. Discussion

### 3.1. ZmNAC17 Coordinates Transcriptional and Post-Transcriptional Regulation of Stalk Development

Plant height development is tightly controlled by phytohormone homeostasis, particularly gibberellin and auxin signaling [5,6,7]. Our study confirms that *ZmNAC17* deficiency leads to a dramatic 98% reduction in bioactive GA3 content, accompanied by decreased GA1 and IAA levels, providing a clear hormonal explanation for the dwarf phenotype of zmnac17 mutants. The upregulation of GA2ox3, which encodes a GA catabolic enzyme, and the elevated expression of GAI-L encoding the DELLA repressor, together with downregulation of GA receptor GID1L2 and F-box protein GID2, suggest that *ZmNAC17* mutation disrupts GA homeostasis primarily through enhanced catabolism and impaired signaling [28,29,30,31]. The partial rescue by exogenous GA3 treatment further supports the causal role of GA deficiency, though the incomplete recovery indicates involvement of additional pathways such as auxin and possibly other hormones. The decreased cell length but increased cell number in *zmnac17-1* provide important insight into the developmental function of *ZmNAC17*. We interpret the elevated cell number as a compensatory developmental response to impaired cell elongation. The severe deficiency in bioactive GA3 and IAA directly constrains cell expansion, while the increased cell number likely reflects prolonged or enhanced meristematic activity that partially offsets the reduced individual cell size. This suggests that *ZmNAC17* primarily regulates internode elongation through cell expansion rather than cell division.

Beyond its role in hormone-mediated cell elongation, our study reveals that *ZmNAC17* fundamentally regulates stalk mechanical strength through a dual-mode molecular mechanism integrating transcriptional activation and protein interaction. As a nuclear-localized transcriptional activator, *ZmNAC17* binds to the promoter of CCoAOMT and activates its transcription, as evidenced by CUT&Tag genome-wide binding profiling, EMSA confirmation of specific DNA-protein interaction, and dual-luciferase reporter validation of promoter activation. This finding is consistent with the established role of NAC transcription factors as master switches of secondary cell wall biosynthesis [32,33,34,35]. In Arabidopsis, SND1 and NST1/3 directly activate downstream MYB46/83 and subsequently lignin biosynthetic genes [33,34]; our results extend this paradigm by demonstrating that *ZmNAC17* can bypass the MYB intermediate and directly activate a key lignin biosynthetic enzyme gene, representing a streamlined regulatory architecture. The activation of lignin biosynthesis correlates with the altered lignin monomer composition observed in transgenic lines. The significant increase in G-type monomer content and the decreased S/G ratio in overexpression lines mechanistically explain the enhanced bending resistance. G-type monomers, due to their unsubstituted C5 position, can form more resistant C–C inter-unit linkages (β-5 and 5-5 bonds), generating highly branched and compact three-dimensional polymer networks that bind more tightly to cellulose microfibrils [32,33,34,35,36,37,38]. Conversely, S-type monomers predominantly form β-O-4 ether bonds, resulting in more linear and flexible lignin structures [32]. The optimization of the S/G ratio rather than mere quantitative increase in total lignin content has important implications for breeding lodging-resistant varieties [37].

The interaction between ZmNAC17 and BGAF1 reveals a previously unrecognized post-transcriptional regulatory layer in lignin metabolism. BGAF1 belongs to dirigent proteins, which are a class of small proteins that control the stereoselective coupling of phenoxy radicals during lignin polymerization, thereby determining the supramolecular structure and mechanical properties of lignin deposits [39,40]. The co-existence of transcriptional activation and protein interaction suggests that *ZmNAC17* operates through a “transcription-polymerization” cooperative network to achieve precise spatiotemporal control of lignin deposition. This mode provides an efficient mechanism to couple gene expressions with metabolic output, ensuring that produced monomers are assembled into structurally optimized polymers. The spatial separation between the nuclear-localized *ZmNAC17* and BGAF1 raises intriguing questions about the cellular dynamics of their interaction. We speculate that their association may occur transiently in the cytoplasm during protein synthesis and trafficking, or alternatively, that unidentified bridging factors or vesicular transport mechanisms mediate their functional communication. Future studies employing live-cell imaging and split-ubiquitin systems could clarify the subcellular context of this interaction.

### 3.2. Pleiotropic Effects and Source-Sink Trade-Offs in ZmNAC17-Mediated Growth Regulation

The phenotypic spectrum of *zmnac17* mutants extends beyond vegetative traits to reproductive development, revealing *ZmNAC17* as a pleiotropic regulator integrating multiple aspects of plant growth. While *zmnac17-1* showed only a mild reduction in ear length and ear weight with unaffected kernel size, *zmnac17-3* exhibited significantly reduced 100-kernel weight and markedly altered grain nutrient composition. These background-dependent differences may reflect the quantitative nature of *ZmNAC17* function and the differential buffering capacity of distinct genetic backgrounds [41]. At the molecular level, such variation could stem from allelic differences in the *ZmNAC17* promoter or coding region between B73 and B104, or from divergent transcript abundance of genetic modifiers and cell wall biosynthetic genes. Additionally, background-specific differences in hormonal baselines, post-translational modification landscapes (e.g., phosphorylation or ubiquitination), or cell wall precursor availability may alter the efficacy of *ZmNAC17*-mediated transcriptional and protein-complex regulation. Elucidating the exact basis will require near-isogenic line analyses combined with comparative transcriptomic and proteomic profiling.

The inverse relationship between stalk mechanical investment and grain carbohydrate accumulation suggests a potential source-sink trade-off mediated by *ZmNAC17*. When stem sink strength is compromised due to reduced lignin deposition and vascular development, assimilates may be redirected toward grain protein synthesis at the expense of starch storage, or alternatively, impaired vascular function may disrupt efficient photosynthate transport to the grain, leading to imbalanced grain filling [41,42,43]. This finding echoes the classical observation that lodging-resistant varieties often exhibit altered grain composition and highlights that *ZmNAC17*-mediated stalk development is physiologically linked to reproductive growth through resource allocation networks.

From a breeding perspective, the “quality-over-quantity” mode of *ZmNAC17* action offers distinct advantages over classical dwarfing genes. Traditional GA-deficient dwarfs such as d1, d3, d8, and d9 suffer from severe developmental and reproductive defects that preclude direct breeding application [3,12,44,45]. In contrast, *ZmNAC17* overexpression enhances stalk mechanical strength primarily by optimizing lignin monomer composition rather than drastically altering plant architecture, and the associated grain quality changes (elevated protein and lysine) may even confer nutritional benefits in certain end-use scenarios [42,46]. The identification of direct target genes and interacting proteins provides multiple molecular handles for precision breeding—such as promoter editing to enhance target gene responsiveness, or allele mining for optimized *ZmNAC17*-Dirigent interaction affinity. However, given the observed pleiotropic effects on ear development, kernel weight, and kernel nutrient composition, as well as the genetic background-dependent phenotypic variation, future field trials under high-density planting conditions will be necessary to systematically evaluate kernel number per ear and overall yield performance in both *ZmNAC17* knockout and overexpression lines, ensuring that enhanced stalk mechanical strength does not come at an unacceptable reproductive cost.

Comparative analysis reveals that the role of *ZmNAC17* as a master regulator of secondary cell wall deposition is evolutionarily conserved with the Arabidopsis SND1/NST1 and rice SNAC1 proteins, which similarly activate downstream MYB transcription factors to control lignin and cellulose biosynthesis [17]. However, our discovery that *ZmNAC17* directly activates lignin biosynthetic genes and physically interacts with cell wall-associated proteins represents a streamlined, maize-specific regulatory architecture that couples transcriptional activation with post-transcriptional polymerization control. From a breeding perspective, it would be interesting to determine whether kernel number per ear is affected by the disruption of ZmNAC17. Furthermore, examining these yield-related traits in ZmNAC17 overexpressors would enhance the significance of the findings [47]. Precision editing of *ZmNAC17*, or its downstream binding motifs, offers a promising strategy to enhance stalk mechanical strength through lignin monomer optimization without severe dwarfing or yield penalty. Such improvements could enable higher planting densities, thereby contributing to field yield increase while maintaining lodging resistance.

### 3.3. Concluding Remarks

In summary, this study establishes *ZmNAC17* as a multifaceted regulator of maize stalk development operating through at least three interconnected pathways: (i) modulation of GA and auxin homeostasis to control cell elongation and plant height; (ii) transcriptional activation of lignin biosynthetic gene *CCoAOMT* to optimize monomer supply; and (iii) physical interaction with BGAF1 to regulate polymerization pattern. The integration of these regulatory layers enables *ZmNAC17* to coordinate hormone signaling, secondary cell wall biosynthesis, and mechanical performance in a coherent developmental program. Future research should focus on evaluating the agronomic performance of *ZmNAC17*-edited lines under high-density planting conditions to validate their practical utility in lodging-resistant breeding.

## 4. Materials and Methods

### 4.1. Plant Materials and Phenotypic Analysis

To validate the function of *ZmNAC17* (Zm00001eb185110), we employed two independent loss-of-function alleles: the EMS-induced *zmnac17-1* mutant in the B73 background, and a newly generated CRISPR/Cas9 mutant (*zmnac17-3*) in the B104 background. *zmnac17-1* was collected from the Qilu Normal College M3 EMS mutant library and was identical to the one used previously [22,48]. B104 is one of the most used inbred lines in maize genetics, due to its high transformation efficiency [49]. In this study, *zmnac17-1* was used for morphological, cellular, and biochemical characterizations, whereas *zmnac17-3* was used for mechanical strength and grain quality analyses.

The CRISPR/Cas9 gene-editing materials were generated by Beijing Bomeixing’ao Technology Co., Ltd., Beijing, China. Three sgRNAs targeting the first exon of *ZmNAC17* were designed: T1 (5′-GATCGAGTCGACGCTGCCAC-3′), T2 (5′-GCAGGGGACGCTCGTCGAGG-3′), and T3 (5′-GGCCTTCCAGTAGCCCGTCC-3′), respectively. The CRISPR construct was transformed into B104 maize immature embryos to generate transgenic plants. The homozygous mutants were identified by PCR using primers F: 5′-GTTCGGAGAGAATCATCGAGTC-3′ and R: 5′-AATGCTGTACGTAACATGCACG-3′, followed by Sanger sequencing. A homozygous mutant carrying a 1 bp deletion at the T2 target site was identified by PCR and Sanger sequencing, resulting in a frameshift mutation and premature stop codon (Figure S5). The positive T0 plants were self-pollinated to produce T1 and T2 generations. For phenotypic and transcriptional analyses, two independent homozygous knockout lines (KO1 and KO2) were examined.

*ZmNAC17* overexpression transgenic lines were generated by Beijing Bomeixing’ao Technology Co., Ltd. In general, the *ZmNAC17* coding sequence was amplified and inserted into the 521 plasmid, furthered by the ZmUBI promoter. The transformation was performed following the standard Agrobacteria-mediated transformation protocol for maize, using B104 immature embryos. Positive transformation events were selected based on kanamycin and bar herbicide resistance. Positive transgenic lines were confirmed with PCR.

For phenotypic analysis, the *zmnac17-1* mutant and the wild-type (B73) of the same M5 family were used. The seeds were planted in the agricultural experimental field of Huangliu Town (108°79′ E, 18°51′ N), Hainan Province, for two years and nursed with standard agricultural cultivation. Plant height and ear height were measured from the ground to the top of the tassel and from the ground to the primary ear, respectively. The long axle and short axle lengths of the third internode were measured using vernier calipers. Stem diameter was calculated as the average value. Mature ear length and width were measured using vernier calipers. Kernel length and kernel width were measured by randomly selecting 10 kernels from the center of the ears. At least 20 individual plants for each genotype were analyzed.

Stalk mechanical strength measurement: Rind penetration resistance and bending force were measured at the R3 stage using a plant stem strength tester (YYD-1B, Zhejiang Top Instrument Co., Ltd., Hangzhou, China). For rind penetration resistance, the probe was inserted perpendicular to the stalk surface at the middle of the third or fourth internode, and the maximum force was recorded. For the bending force, the third internode was placed on two support points, and the maximum load at breaking was recorded with a loading speed of 5 mm/min. At least 15 individual plants for each genotype were analyzed.

Ear and kernel traits of B104 plants were measured using the same methods as described for B73. Kernel nutritional quality analysis (soluble protein, starch, lysine, carotenoid, and total protein contents) was performed on dried kernels ground to fine powder. Soluble protein, starch, lysine, and carotenoid contents were determined using commercial kits (Suzhou Grace Bio-technology Co., Ltd., Suzhou, China) following the manufacturer’s protocols. Total protein content was determined by the Kjeldahl method according to standard protocols.

### 4.2. Tissue Staining and Microscopic Observation

Stem tissues were collected from the sixth internode and male stems at the tasseling stage, respectively. Cut into 2–4 mm thick sections and then immersed in 4% paraformaldehyde. Vacuumized for 30 min and fixed at 4 °C for 48 h. Dehydrated in a graded ethanol series, soaked in a graded xylene series (xylene: pure ethanol, 1:3, 1:1, 3:1, *v*/*v*) for 1 h and 100% (*v*/*v*) xylene for 1 h (two times repeated), and then infiltrated with liquid paraffin at 75 °C. Paraffin sections were generated with a paraffin microtome (RM2235, Leica Microsystems Trading Co., Ltd., Shanghai, China). Stained with 1% toluidine blue solution and observed under a microscope (DM6B LEICA). Cell length, cell area, and vascular bundle area were measured using ImageJ software (version 1.53t, National Institutes of Health, Bethesda, MD, USA).

### 4.3. Measurement of Cellulose and Lignin Content

Maize stalk lodging predominantly occurs at the third to sixth internodes during the grain-filling stage, when the increasing ear weight imposes maximal mechanical load on the lower stalk [23,24]. The sixth internode at one week after pollination (approximately R1–R2 stage) represents a critical developmental window during which secondary cell wall biosynthesis, including cellulose and lignin deposition, is actively proceeding and largely determines the final mechanical strength of the mature stalk [24,25]. Therefore, this stage and internode position are widely adopted as reliable indicators for evaluating lodging resistance in maize. The sixth internode collected one week after pollination was dried and crushed with six biological replicates. The G0715W kit was bought from Suzhou Grace Bio-technology Co., Ltd., Suzhou, China, to measure cellulose content. The protocols were generally the same as for lignin. OD values were measured at 620 nm and 460 nm wavelength using a UV spectrophotometer, respectively. The contents of cellulose were calculated according to the standard curve. The G0708W kit was bought from Suzhou Grace Bio-technology Co., Ltd., Suzhou, China. 1.5 mg of drying material was added to 1.5 mL 80% ethanol, vortexed, and incubated at 50 °C for 20 min, centrifuged at 12,000 rpm for 10 min, and the supernatant was discarded. This was repeated twice. Precipitation was dried at 95 °C. Reagent 1 was added to the precipitation, incubated at 50 °C for 2 h, then reagent 2 and reagent 3 were added, and centrifuged at 500 rpm for 5 min. OD value was measured at 280 nm. Lignin content was calculated according to the standard curve.

Lignin monomer composition analysis: Lignin monomers were analyzed by alkaline CuO oxidation combined with high-performance liquid chromatography (HPLC). Approximately 0.5 g of dried and powdered stem tissue (third internode) was subjected to microwave digestion with CuO and NaOH. The oxidation products were extracted with ethyl acetate, dried, and resuspended in 1.5% acetic acid-acetonitrile solution. HPLC analysis was performed on an Agilent 1260 system with a GOLD-C18 column. Syringaldehyde (S-type) and vanillin (G-type) were quantified by the external standard method, and the S/G ratio was calculated.

### 4.4. Determination of Endogenous Phytohormone Content

The endogenous phytohormone content measurement was performed by Suzhou Grace Bio-technology Co., Ltd., Suzhou, China. Fresh internode tissue was frozen with liquid nitrogen, then stored at −80 °C. In total, 500 mg tissue was added into 1 mL pre-cooled 85% methanol aqueous solution, ground into powder, extracted at 4 °C for 12 h, and centrifuged at 13,000 rpm for 5 min. The supernatant was added to 1 mL of the extraction solution and shaken for 10 min, concentrated to 0.5 mL using nitrogen blowing. The concentrated solution was adjusted to pH 3–4 with formic acid–water. Ethyl acetate was used for 3-time repeated extractions. Purified by the C18 solid phase extraction column, eluted with 3 mL methanol, and dried using blown nitrogen. Dissolved in 300 µL methanol, then filtered through a 0.22 µm nylon needle filter and collected into the sample bottle to be tested. The contents of endogenous hormones were determined by liquid chromatography tandem mass spectrometry (LC-MS). Standard Gibberellin A1 (ZTR-G377495, HPLC ≥ 98.0%) was bought from CYMIT QUÍMICA S.L., Barcelona, Spain. Standard Gibberellin A3 (b20187 HPLC ≥ 90.0%), standard Gibberellin A4 (B22408, HPLC ≥ 98.0%), and standard IAA (b21810 HPLC ≥ 98.0%) were bought from Shanghai Yuanye Bio-Technology Co., Ltd., Shanghai, China.

### 4.5. GA3 Treatments

Maize seeds were sown in a seedling tray and grown for 7 days, then transplanted into the field. GA3 (G8040, HPLC ≥ 90.0%) was bought from Beijing Solarbio Science & Technology Co., Ltd., Beijing, China. GA3 was dissolved in ethanol as suggested to 50 mg/mL, then diluted with distilled water into 10 µM, 100 µM, 500 µM, and 1000 µM, respectively. The diluted GA3 solution was sprayed on the leaves of plants at the V6 stage. The control group was sprayed with the same amount of distilled water (0 µM GA3). Photos were taken, and plant height was measured at the VT stage.

### 4.6. RNA Sequencing and Data Analysis

For *zmnac17-1* and B73, RNA-seq analysis was performed by Metware Biotechnology Inc. (Metware), Wuhan, China. The sixth internodes of plants grown for 40 days were harvested and frozen in liquid nitrogen. A total of five biological replicates were used, with 1 µg of qualified RNA per sample. Sequencing libraries were generated using the NEB Next Ultra TM RNA library prep kit for Illumina (New England Biolabs, Ipswich, MA, USA). The library quality was assessed on an Agilent Bioanalyzer 2100 system. Illumina sequencing was performed using the NovaSeq 6000 System. After removing reads containing adapters or poly-N and low-quality reads (q-value ≤ 20), the paired-end clean reads were aligned to the B73 reference genome (RefGen_v5) using the default parameters of HISAT v2.1.0 software. Fragments per kilobase pair of exons per million fragments mapped (FPKM) were used to normalize gene expression values. The differential expression analysis was performed using DESeq2. The resulting *p*-values were adjusted using Benjamini and Hochberg’s approach for controlling the false discovery rate (FDR). Genes with Log2 fold-change (Log2FC) ≥ 1 (up-regulated) or Log2FC ≤ −1 (down-regulated) and FDR < 0.01 were considered as differentially expressed genes (DEGs).

For *ZmNAC17*-HA overexpression (OE) lines and B104, RNA-seq was performed by Wuhan Biorun Bio-tech Co., Ltd., Wuhan, China. The third internodes at the mid-grain-filling stage were harvested and frozen in liquid nitrogen. Three biological replicates were used for each genotype (OE lines and wild-type B104). RNA extraction, library construction, sequencing, and data analysis were performed by Metware Biotechnology Inc. (Metware), Wuhan, China, using the same protocols and parameters as described above for the *zmnac17-1* RNA-seq. Genes with |Log2FC| ≥ 1 and FDR < 0.01 were considered as differentially expressed genes.

CUT&Tag analysis was performed by Wuhan Biorun Bio-tech Co., Ltd., Wuhan, China. CUT&Tag was performed using *ZmNAC17*-HA overexpression plants [50]. Nuclei were isolated and bound to ConA magnetic beads, incubated with anti-HA antibody and protein A-Tn5 fusion complex, and tagmented. Libraries were constructed and sequenced on the Illumina NovaSeq 6000 platform. Data analysis was performed using Trimmomatic v0.39 [51,52], BWA v0.7.17 [53], MACS2 v2.2.7.1 [54], and HOMER v4.11. Peaks located within 2 kb upstream of transcription start sites with fold enrichment ≥ 4 were considered significant.

EMSA was performed by Wuhan Biorun Bio-tech Co., Ltd., Wuhan, China. The MBP-*ZmNAC17* fusion protein was expressed in *E. coli* and purified. Biotin-labeled probes containing the predicted cis-element were synthesized. Protein-DNA binding reactions were performed and separated on 6% native polyacrylamide gels, followed by detection using streptavidin-HRP.

A dual-luciferase reporter assay was performed by Wuhan Biorun Bio-tech Co., Ltd., Wuhan, China. The target gene promoter was cloned into the pGreenII 0800-LUC reporter vector, and *ZmNAC17* was cloned into the pGreenII-62SK effector vector. Constructs were co-transformed into maize protoplasts, and LUC/REN ratios were measured using the dual-Luciferase Reporter Assay System [41].

IP-MS and protein interaction validation were performed by Wuhan Biorun Bio-tech Co., Ltd., Wuhan, China. For IP-MS, total protein from *ZmNAC17*-HA overexpression plants was immunoprecipitated with anti-HA antibody, and co-precipitated proteins were identified by mass spectrometry. For Co-IP, FLAG-*ZmNAC17*-GFP and the candidate protein were co-expressed in maize protoplasts, and immunoprecipitation was performed with GFP-Nanoab-Agarose. For GST pull-down, MBP-*ZmNAC17* and GST-candidate protein were expressed in E. coli and incubated with Glutathione Agarose beads.

### 4.7. Quantitative Real-Time PCR (qRT-PCR) Validation of the DEGs

DEGs were randomly selected for qRT-PCR validation (Figure S4). The specific primers for qRT-PCR can be found in Table S3. The reaction was performed using SYBR Green Pro Taq (Accurate Biotechnology, Co., Ltd., Wuhan, China) and the CFX96 real-time PCR detection system (Bio-Rad, Hercules, CA, USA). The thermal cycling program consisted of an initial denaturation at 95 °C for 30 s, followed by 40 cycles of denaturation at 95 °C for 5 s and annealing/extension at 60 °C for 30 s. A dissociation stage (melting curve analysis) was included to verify amplification specificity. The cycle threshold (CT) and 2^−∆∆Ct^ method were utilized to assess relative transcript levels, which were normalized using Actin as an internal control. The reaction was performed with three biological replicates and three technical replicates.

### 4.8. Statistical Analysis

Student’s *t*-test was performed using SPSS (version 20.0), and figures were drawn using GraphPad Prism 8.0. The data were presented as mean ± SD. * (*p* < 0.05), ** (*p* < 0.01) and *** (*p* < 0.001) indicate significant differences. ns indicates no significant change.

## Data Availability

The raw sequence data reported in this study were deposited in the Genome Sequence Archive [55], National Genomics Data Center [56], China National Center for Bioinformation/Beijing Institute of Genomics, Chinese Academy of Sciences (GSA: CRA015622), and are publicly accessible at https://ngdc.cncb.ac.cn/gsa (accessed on 26 September 2024).

## Supplementary Materials

The following supporting information can be downloaded at: https://www.mdpi.com/article/10.3390/plants15121814/s1, Figure S1: Differentially expressed genes between WT and *zmnac17-1*; Figure S2: The significantly enriched GO terms of the DEGs between WT and *zmnac17-1*; Figure S3: The significantly enriched KEGG pathways of the DEGs between WT and *zmnac17-1*; Figure S4: The quantitative verification of RNA-seq data using qRT-PCR. Primers can be found in Table S3. Three biological replicates were used. The data presented are means ± SD and statistically calculated by Student’s unpaired *t*-test (* *p* < 0.05, ** *p* < 0.01, *** *p* < 0.001); Figure S5: Genotyping of *zmnac17-3*. Exons are labeled E1, E2, and E3. T1 and T2 indicate guide RNAs targeting two distinct sites. Table S1: The DEGs related to other phytohormone synthesis signaling; Table S2: The DEGs related to TFs; Table S3. Primers for qRT-PCR validation.
