# Supplementary material for: ZmNAC17 Integrates Transcriptional and Protein Interaction Networks to Regulate Maize Stalk Architecture
Source: Plants (Basel). 2026 Jun 12;15(12):1814. doi: 10.3390/plants15121814 (PMC13306793; doi:10.3390/plants15121814)
Supplement: Supplementary file 1 [file plants-15-01814-s001.zip › plants-4314086-Supplementary Materials.pdf]

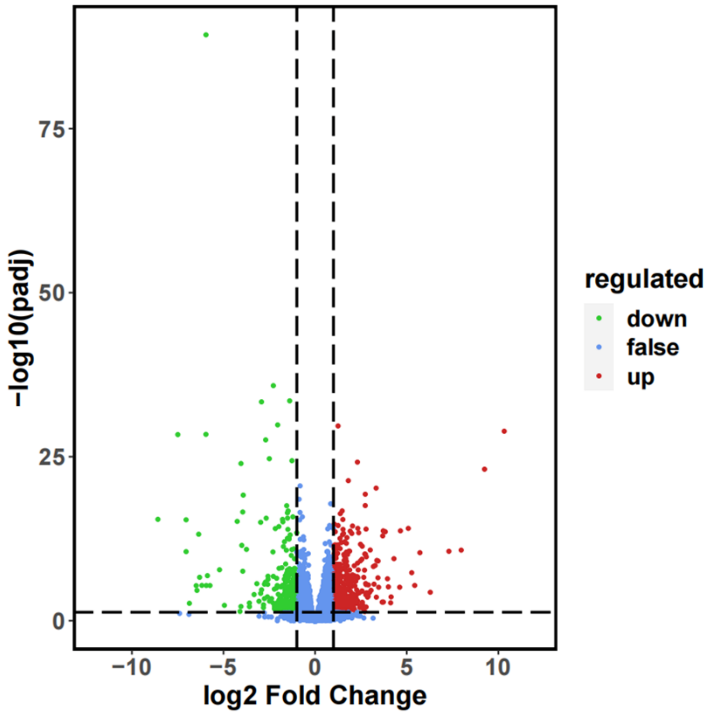

Figure S1. Differentially expressed genes between B73 and *zmnac17-1*.

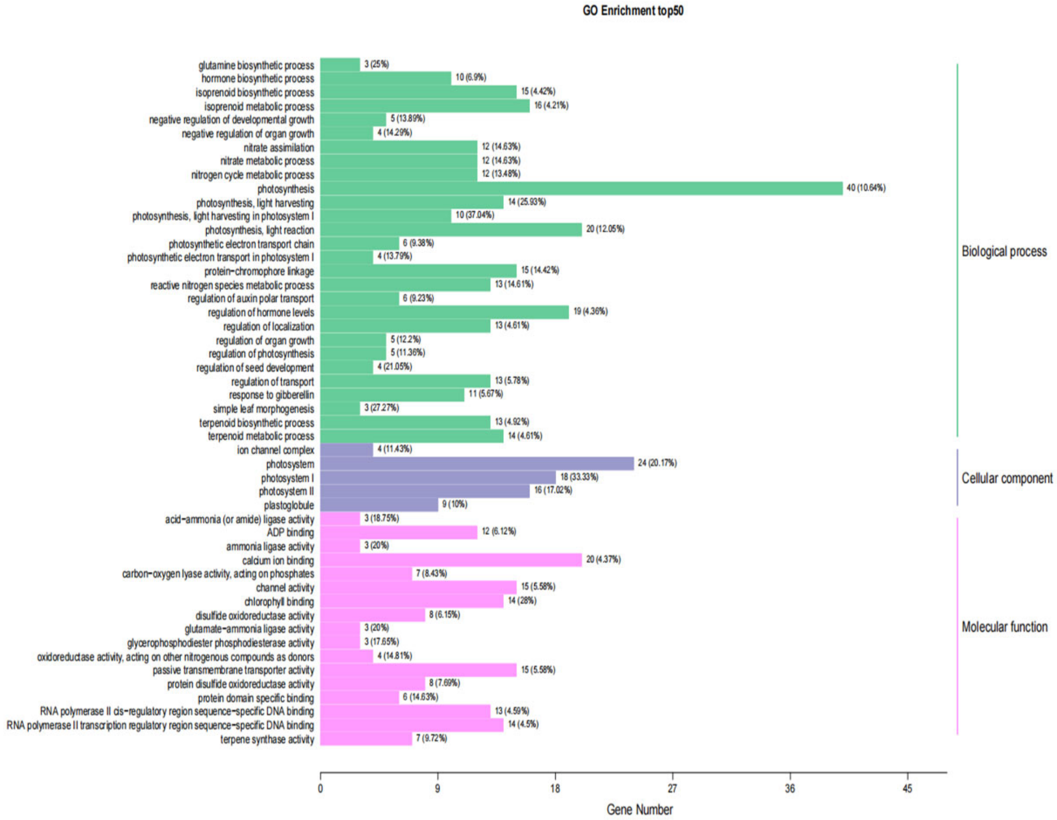

Figure S2. The significantly enriched GO terms of the DEGs between B73 and *zmnac17-1*.

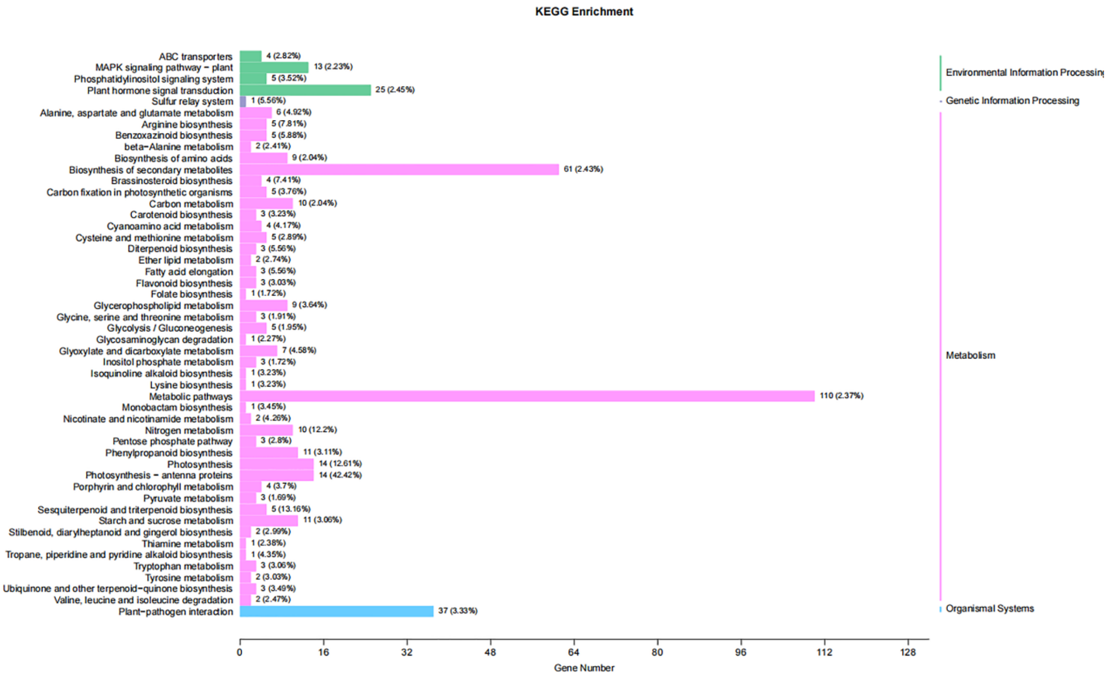

Figure S3. The significantly enriched KEGG pathways of the DEGs between B73 and *zmnac17-1*.

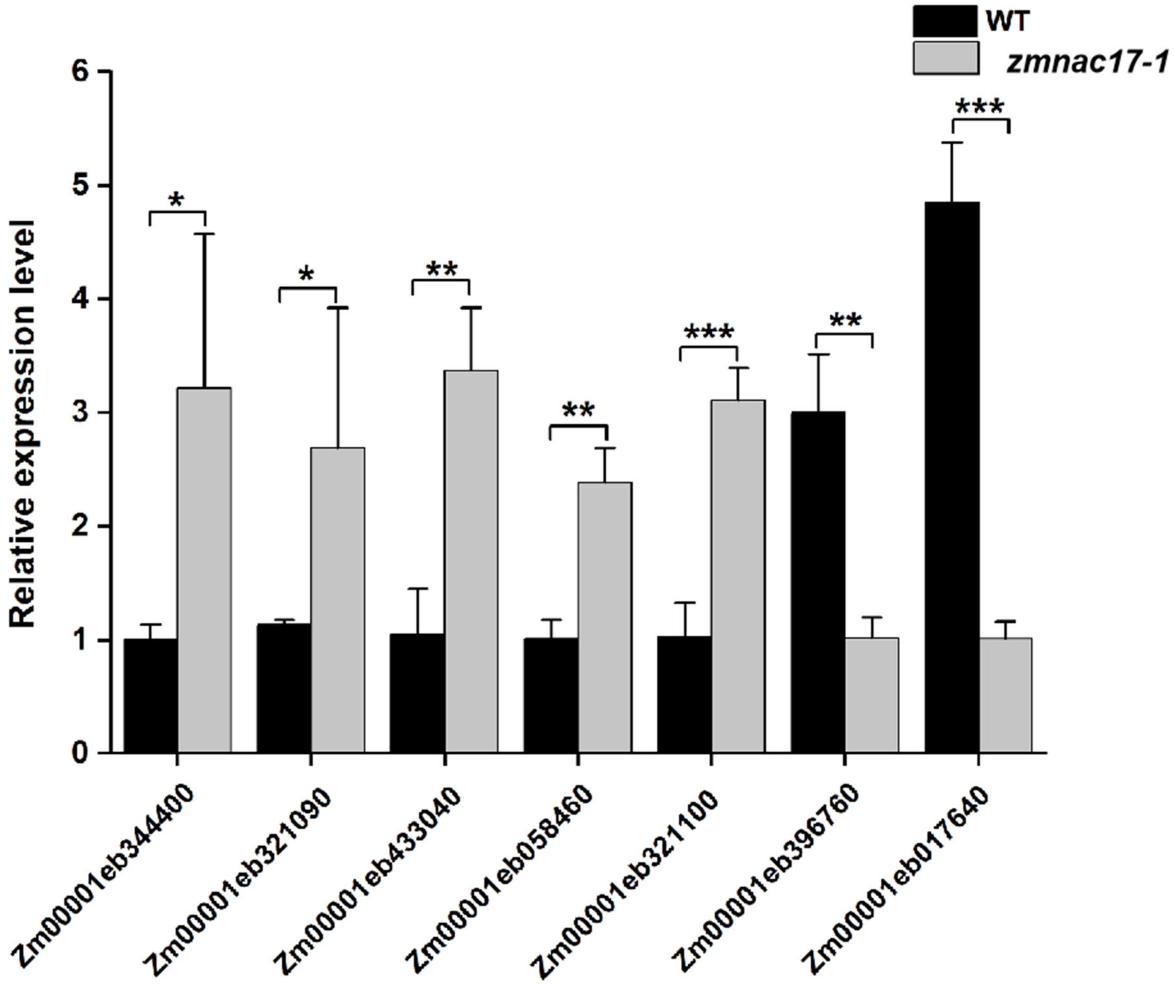

Figure S4. The quantitative verification of RNA-seq data using qRT-PCR. Primers can be found in Table S3. Three biological replicates were used. The data presented are means±SD and statistically calculated by student's unpaid t-test (* $p < 0.05$, ** $p < 0.01$, *** $p < 0.001$).

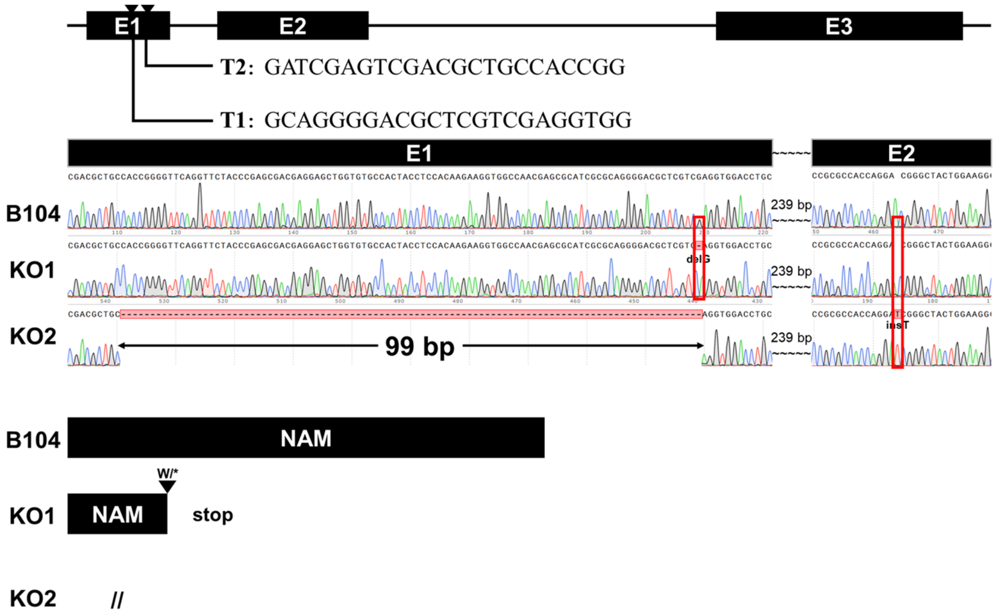

Figure S5. Genotyping of *zmnac17-3*. Exons are labeled E1, E2, and E3. T1 and T2 indicate guide RNAs targeting two distinct sites.

Table S1. The DEGs related to other phytohormones synthesis signaling.

Phytohormone	Gene ID	Protein	Log2FC	pvalue
BR	Zm00001eb017640	flavonoid 3-monooxygenase	-1.097399747	3.11E-07
	Zm00001eb073640	cytochrome P450 87A3-like	-1.215498412	1.96E-06
	Zm00001eb100210	cytochrome P450 monooxygenase CYP92A	1.212042063	3.97E-08
	Zm00001eb218960	cytochrome P450 87A3	1.683124214	5.96E-07
	Zm00001eb184670	probable LRR receptor-like serine/threonine-protein kinase	1.520778051	0.002078961
	Zm00001eb290700	uncharacterized protein LOC100275543 isoform X3	1.195394671	1.71E-05
	Zm00001eb309030	brassinosteroid LRR receptor kinase BRL1	1.72877327	1.34E-06
CK	Zm00001eb074370	uncharacterized protein LOC100273251	2.331856426	0.002768376
	Zm00001eb058460	G2-like transcription factor, partial	1.442579784	1.31E-10
	Zm00001eb067190	uncharacterized protein LOC103645799 isoform X2	1.240720847	1.12E-09
	Zm00001eb118010	myb-like DNA-binding domain, SHAQKYF class family protein	1.289428271	8.65E-13
	Zm00001eb291140	myb-related protein 2	1.063653234	6.20E-05
	Zm00001eb406610	Putative MYB DNA-binding domain superfamily protein	3.649984991	1.60E-15
	Zm00001eb433040	transfactor isoform X2	3.017296382	2.63E-19
ABA	Zm00001eb321780	uncharacterized protein LOC100272785	-1.214121153	0.001853606
	Zm00001eb170400	NAD(P)-binding Rossmann-fold superfamily protein	1.376794301	0.000151744
	Zm00001eb307050	uncharacterized protein LOC103632342	-1.001289779	0.000923286
	Zm00001eb383610	ankyrin repeat domain-containing protein 28	1.93917491	3.45E-07
ethylene	Zm00001eb328310	unknown	-1.162028386	1.31E-08
	Zm00001eb016230	uncharacterized LOC100279249	-1.457654079	0.001067838
	Zm00001eb314610	uncharacterized protein LOC100382364	1.178943137	1.79E-12
	Zm00001eb097580	bifunctional aspartokinase/homoserine dehydrogenase 2, chloroplastic isoform X1	-1.00098968	1.18E-05
	Zm00001eb332260	uncharacterized protein LOC100193096	1.120495843	1.02E-05
	Zm00001eb331080	protein ETHYLENE INSENSITIVE 3	-1.091910698	5.96E-09
JA	Zm00001eb082050	12-oxo-phytodienoic acid reductase 5	1.779890897	1.83E-06
	Zm00001eb223590	protein TIFY 10b	1.957415859	0.000236471
SA	Zm00001eb420190	primary amine oxidase	-2.590927296	1.01E-11
	Zm00001eb147220	Basic leucine zipper protein	1.972295984	8.21E-15

Table S2. The DEGs related to TFs.

TF	Gene ID	Protein	Log2FC	pvalue
NAC	Zm00001eb002660	uncharacterized protein LOC100501100	1.122214084	0.004209104
	Zm00001eb036920	NAC transcription factor, partial	1.082724597	1.83E-20
	Zm00001eb076470	secondary wall NAC transcription factor 4	1.07879337	0.001364405
	Zm00001eb149810	NAC protein isoform 1	1.313366831	0.001780421
	Zm00001eb175500	NAC domain-containing protein 68	1.081611086	1.11E-05
	Zm00001eb317890	putative NAC domain transcription factor superfamily protein isoform X1	1.198109341	2.64E-13
	Zm00001eb359950	Putative NAC domain transcription factor superfamily protein	1.055676278	0.00707591
	Zm00001eb369060	uncharacterized protein LOC100381829	1.58428072	6.69E-05
MADS	Zm00001eb057540	Agamous-like MADS-box protein AGL8	-1.653103149	1.45E-31
	Zm00001eb057560	MADS24	-2.474272852	9.06E-07
	Zm00001eb118120	zea apetala homolog 1	-2.348954001	1.06E-18
	Zm00001eb214740	MADS31	-2.902028066	9.98E-06
	Zm00001eb214750	m15 protein isoform X2	-2.604262241	1.82E-11
	Zm00001eb253580	Agamous-like MADS-box protein AGL16	-1.266932063	0.001172286
	Zm00001eb298680	MADS3	-2.768366743	3.97E-06
	Zm00001eb403750	MADS1	1.26300105	1.31E-08
GRAP-G2-like	Zm00001eb058460	G2-like transcription factor, partial	1.442579784	1.31E-10
	Zm00001eb067190	uncharacterized protein LOC103645799 isoform X2	1.240720847	1.12E-09
	Zm00001eb118010	myb-like DNA-binding domain	1.289428271	8.65E-13
	Zm00001eb291140	myb-related protein 2	1.063653234	6.20E-05
	Zm00001eb406610	Putative MYB DNA-binding domain superfamily protein	3.649984991	1.60E-15
	Zm00001eb433040	transfactor isoform X2	3.017296382	2.63E-19
bZIP	Zm00001eb033630	bZIP protein	1.039404019	9.90E-05
	Zm00001eb147220	Basic leucine zipper protein	1.972295984	8.21E-15
	Zm00001eb238570	bZIP transcription factor family protein	1.733846146	4.08E-17
	Zm00001eb389660	light-inducible protein CPRF-2 isoform X1	1.68824592	8.68E-08
	Zm00001eb399090	transcription factor HBP-1a isoform X1	-7.873260757	3.62E-15
AP2/ERF	Zm00001eb168040	Putative AP2/EREBP transcription factor	3.272212614	5.25E-06
	Zm00001eb285760	unknown	-1.145179105	0.008351314
	Zm00001eb299300	putative AP2/EREBP transcription factor superfamily protein isoform X1	-1.047821373	0.003120727
	Zm00001eb387280	glossy15 isoform X2	2.172683157	1.09E-11

	Zm00001eb389400	heat shock complementing factor 1	3.921436897	1.93E-07
MYB	Zm00001eb091300	Transcription repressor MYB	-1.06493508	0.000123335
	Zm00001eb121290	transcription factor RAX2	1.84007108	0.000172218
	Zm00001eb138920	transcription factor MYB8	-1.1688523	0.000125295
	Zm00001eb213800	transcription factor MYB15 isoform X2	2.67035213	7.84E-11
	Zm00001eb332030	myb-related protein Hv1	1.143950326	6.24E-11
	Zm00001eb366540	myb-related protein 308	1.242745487	5.08E-08
MYB-related	Zm00001eb064900	MYB-related transcription factor, partial	1.099368864	8.06E-15
	Zm00001eb072870	unknown	1.520471849	1.21E-05
	Zm00001eb344000	SANT/MYB protein	-1.501711726	0.00049036
	Zm00001eb416380	uncharacterized protein LOC110467540	-4.029510122	0.00040551
bHLH	Zm00001eb101880	transcription factor bHLH130	1.373262392	4.51E-06
	Zm00001eb188380	putative HLH DNA-binding domain superfamily protein	1.311825646	4.03E-12
	Zm00001eb252100	Transcription factor bHLH63	1.533700558	8.10E-09
C2H2	Zm00001eb080470	uncharacterized protein LOC109944164	-1.090041721	0.004139897
	Zm00001eb320600	INDETERMINATE-related protein 7	-1.06247201	1.00E-07
	Zm00001eb339510	zinc finger protein NUTCRACKER	1.803869119	0.002915962
GRAS	Zm00001eb344400	DELLA protein GAI-like	1.762908829	0.000500732

Table S3. Primers for qRT-PCR validation.

Gene Name	Sequence (5'-3')
Actin-F	GTCCATGAGGCCACGTACAA
Actin-R	CCGGACCAGTTTCGTCATA
Zm00001eb396760-F	CTGCCCGGAATGTCTCAAAG
Zm00001eb396760-R	ACAGTCACAATCCAGACGGC
Zm00001eb321100-F	GTCAGCGCCAAGAGACTTGT
Zm00001eb321100-R	GCTACAGCTACACGCGAGG
Zm00001eb017640-F	CTACGATCCTGGGCAACCTG
Zm00001eb017640-R	TCACAGGACAGCATACAGGC
Zm00001eb344400-F	TGTCTGAGGCACGCAAGATT
Zm00001eb344400-R	GCAGTGGAGTAGGCGAACTT
Zm00001eb321090-F	TCAGGGAGCTCCTGCAAATG
Zm00001eb321090-R	GTAGTGGCAGCACGACGTT
Zm00001eb433040-F	TTCACTCGAGCTGCCATTCC
Zm00001eb433040-R	GGACAGCGGGTTGTCCATAA
Zm00001eb058460-F	CGAAGCAGATCAGGGAGGTG
Zm00001eb058460-R	CTGATGAGGAGGAGCCCAGA
